# Pediatric Mental Health Needs, Unmet Care, and Disaster-Related Displacement

**DOI:** 10.1001/jamanetworkopen.2026.4922

**Published:** 2026-04-14

**Authors:** Joniqua N. Ceasar, Keven I. Cabrera, David Mandell

**Affiliations:** 1 National Clinician Scholars Program, Perelman School of Medicine, University of Pennsylvania, Philadelphia; 2 Department of Psychiatry, University of Pennsylvania, Philadelphia; 3 PolicyLab, Children’s Hospital of Philadelphia, Philadelphia, Pennsylvania; 4 Department of Emergency Medicine, Children’s Hospital of Philadelphia, Philadelphia, Pennsylvania

## Abstract

**Importance:**

Extreme weather disasters leave behind long-lasting mental and physical health problems. Displacement related to climate change disproportionately impacts children, whose developmental stage leaves them particularly at risk to its devastating effects.

**Objective:**

To compare pediatric mental health needs and care for those in need between displaced households and other households.

**Design, Setting, and Participants:**

This repeated cross-sectional analysis with multivariable logistic regression models used data from the US Census Household Pulse Survey, a nationally representative dataset, from June 1, 2023, through September 31, 2024. The study included biweekly, individual-level data reported by individual adults for all members of their household.

**Exposure:**

Experience of displacement in the past year due to a natural disaster.

**Main Outcomes and Measures:**

Mental health needs were defined based on response to a question asking whether any children in the household needed mental health treatment, such as counseling or medication, in the past 4 weeks. Then the likelihood of receiving mental health treatment was examined for those who reported mental health needs.

**Results:**

Of the 324 466 households with children identified during the study period, 277 081 respondents (mean [SD) age, 42.7 [0.05) years; 162 288 [58.6%] female) were included in the analytic sample. This sample represents approximately 35 million US households with children. Among households with children, those that experienced displacement due to natural disaster had a significantly higher chance of reporting that the children needed mental health counseling or medication (odds ratio, 1.29; 95% CI, 1.12-1.48; *P* < .001). Among households with reported pediatric mental health needs, displaced households were more likely to report inadequate or no treatment, even after controlling for socioeconomic hardships (odds ratio, 0.55; 95% CI, 0.38-0.66; *P* < .001).

**Conclusions and Relevance:**

The cross-sectional study found that displaced children experience more unmet mental health needs after natural disasters compared with nondisplaced children, which may negatively impact their long-term development. As climate change progresses, targeted research is urgently needed to understand how best to meet these needs.

## Key Points

**Question:**

What is the pediatric mental health burden among US households with children who have experienced disaster-related displacement?

**Findings:**

In this cross-sectional study of 277 081 households with children, displaced households reported higher odds of needing mental health counseling or medication than nondisplaced households. Among those who needed mental health care, displaced households were more likely than other households to report inadequate treatment even after controlling for socioeconomic hardships.

**Meaning:**

This study’s findings suggest that health care professionals, community leaders, and policymakers should prioritize children’s well-being in disaster preparedness, response, and recovery planning to ensure they have access to mental health resources amid extreme weather events.

## Teaser

This cross-sectional study of households with children examines the association of displacement with pediatric mental health needs and receipt of care.

## Introduction

Extreme weather disasters, such as wildfires, tornadoes, hurricanes, and floods, cause immediate destruction and leave behind long-lasting mental and physical health problems.^1^ Worldwide, the number of disaster displacements in 2023 was nearly double the annual average of the past decade, which aligns with the surge in natural disasters.^2^ Although displacements historically have disproportionately occurred in lower-income countries and been related to conflict, the US faces an increasing displacement crisis.^3,4,5^ More than 11 million people were displaced within the US in 2024, representing approximately one-quarter of all disaster displacements globally.^2^

The increased frequency and intensity of extreme climate or weather events will expose more children to displacement and cascading adverse childhood experiences.^3,4,6^ Disaster displacement is an understudied social determinant of health. Displacement is a short-term stressor layered on long-term social risk, so these events exacerbate existing health needs. Emerging evidence shows that people with minoritized race and ethnicity and those with lower incomes are more likely to report displacement related to natural disasters.^7^ Climate-related events and related displacement can lead to mental health problems and subsequent poor academic performance, unemployment, increased health care costs, and strained social services.^8,9,10,11^

The American Academy of Pediatrics recommends integrating mental health considerations throughout all disaster phases, including preparedness, response, and recovery.^12^ Limited research has assessed the extent of mental health needs among displaced households with children and their ability to obtain treatment, making this planning difficult. In this study, we use nationally representative data to examine the association of displacement with pediatric mental health needs and receipt of care.

## Methods

In accordance with the Common Rule and the University of Pennsylvania Institutional Review Board guidelines, this study was exempt from ethics review and informed consent requirement because deidentified data were used. We followed the Strengthening the Reporting of Observational Studies in Epidemiology (STROBE) reporting guideline.

### Data

We used the US Census Bureau’s publicly available Household Pulse Survey (HPS), which launched in 2020 and addresses emergency socioeconomic issues. Households were randomly selected from the US Census Bureau’s Master Address File. Each household designated one resident to complete a 20-minute questionnaire. The HPS features survey responses from more than 2 million households but overall has a low response rate, as previously described.^7^ Nonresponse bias is minimized by using survey weights that provide nationally representative estimates.

The exposure of interest was displacement based on questions introduced in December 2022. The outcomes were pediatric mental health needs and treatment receipt based on questions introduced in June 2023. We conducted a cross-sectional complete case analysis for the exposures, outcomes, and covariates, which resulted in an analytic sample for June 1, 2023, through September 31, 2024, drawing from 15 waves of data collection. The derivation of the analytic sample is shown in the eFigure in Supplement 1.

We identified state-level mental health practitioner availability from the December 2024 Health Professional Shortage Area report published by the Health Resources and Services Administration (HRSA).^13^ The quarterly report provides the percentage of mental health care needs met within each state, calculated by HRSA as the number of psychiatrists available to serve a population divided by the number needed to eliminate the shortage, using a population to psychiatrist ratio of 30 000:1 (20 000:1 for areas with high needs). We used this metric to create a proxy for state-level mental health practitioner scarcity.

### Covariates

Model covariates included adult respondent sociodemographic characteristics, disability status, insurance type, experience of depression and/or anxiety, overall household characteristics, and mental health practitioner scarcity. Sociodemographic factors included survey respondent age, sex assigned at birth, self-reported race and ethnicity, educational status, employment status, recent household job loss, marital status, disability status, and insurance type. Race and ethnicity categories included Hispanic, non-Hispanic Asian, non-Hispanic Black, non-Hispanic White, and non-Hispanic multiracial or other race, including American Indian or Alaska Native, Native Hawaiian, Chamorro, Samoan, and Other Pacific Islander. Mental health characteristics included symptoms of adult respondent depression and anxiety using the Generalized Anxiety Disorder 2 and Patient Health Questionnaire 2 questionnaires. Household characteristics included household income, total household size, and number of children in household. Material hardships, which may influence both displacement and mental health, included housing security (combined indicator of housing tenure and payment status), food insufficiency, and energy insecurity. More detailed information about the HPS instrument and variable categorization can be found in eTable 1 in Supplement 1.

To account for state-level mental health care availability, we used HRSA’s percentage of need met metric from the December 2024 Health Professional Shortage Area report and transformed it into a measure of percent unmet need with the following formula: (100 – percentage of need met). States were categorized into quartiles of unmet need: lowest, low to moderate, moderate to high, and highest unmet need. Data were not available for Vermont.

### Exposure

Our exposure was displacement due to disaster, defined as a yes or no in response to the question, “In the past year, were you displaced from your home because of a natural disaster?” Nondisplaced households were the reference group for all analyses.

### Outcomes

We assessed 2 outcomes regarding mental health needs and receipt of mental health care. For reported need, we created a binary outcome variable based on responses to the question, “During the last 4 weeks, did any children in your household need mental health treatment? Mental health treatment includes health services like counseling or medication.” Those who answered “yes, all children needed mental health treatment” or “yes, some children needed mental health treatment” were coded as yes, whereas those who answered “no, none of the children needed mental health treatment” were coded as no. For receipt of care, the outcome was defined by the response to the question, “Did the children who needed mental health treatment receive it?” We created a binary outcome variable based on 3 survey answers: (1) none of the children who needed treatment received it, (2) only some of the children who needed treatment received it, and (3) all children who needed mental health treatment received it. Respondents who answered “none or only some children received treatment” were coded as 0 (incomplete treatment), and those who answered “all children received treatment” were coded as 1 (complete treatment).

### Statistical Analysis

We assessed 2 survey-weighted multivariable regression logistic models. We assessed collinearity to confirm model covariate inclusion and concluded no serious multicollinearity as confirmed by mean variance inflation factor values below 2. For each model, we calculated descriptive statistics to summarize differences between groups, stratified by exposure. Statistical significance was examined using linear regression for continuous variables and χ^2^ analyses for categorical variables. SEs were calculated using Taylor series linearization to account for the complex survey design, and 95% CIs were derived from design-based SEs. Statistical significance was defined as a 2-sided *P* < .05. All data, including survey weights, were publicly available.^14^ We used Stata, version 18 (StataCorp) for all analyses.

#### Model 1: Report of Mental Health Needs

For our regression model assessing the exposure of displacement and outcome of pediatric mental health needs, covariates included age, race, ethnicity, marital status, employment status, recent household job loss, disability status, educational status, household income, ownership and mortgage or rent payment status, household size, number of children in household, food insufficiency, energy insecurity, adult anxiety and/or depression, and insurance type. The model adjusted for state fixed effects. We also estimated adjusted predicted probabilities of mental health need by displacement status.

#### Model 2: Receipt of Mental Health Care for Those in Need

We created a subsample that included only households who reported yes to the question about pediatric mental health care needs and assessed their receipt of mental health treatment. Displacement again served as our exposure, with nondisplaced households as the reference group. The regression model included state fixed effects and model 1 covariates with the addition of mental health provider scarcity.

#### Sensitivity Analyses

We reclassified the binary outcome using a less restrictive definition, coding households in which no children received mental health treatment as 0 and those in which some or all children received treatment as 1. We then reestimated model 2 to assess the robustness of findings related to mental health care receipt. eTable 2 in Supplement 1 showcases the main models and sensitivity analyses.

#### Supplemental Analyses

We calculated descriptive statistics that compared households with children included in the analytic sample (n = 227 081) with those excluded due to missing data (n = 47 385). Differences in demographics, socioeconomic characteristics, and outcomes of interest were examined to assess potential selection bias introduced by complete case analysis methods.

Using model 2, we tested for moderation by household income and by the shortage in mental health professionals using interaction terms due to prior literature^15,16^ that these are significant drivers of mental health care receipt. Specifically, we included interaction terms between displacement status and income category (displacement × income) and between displacement and quartiles of unmet need (displacement × unmet need) in fully adjusted logistic regression models.

## Results

### Pediatric Mental Health Needs

Of the 324 466 households with children identified during the study period, 277 081 (mean [SD] age, 44.9 [0.02] years; 162 288 [58.6%] female and 114 793 [41.4%] male; 30 746 [11.1%] Hispanic, 15 038 [5.43%] non-Hispanic Asian, 24 157 [8.7%] non-Hispanic Black, 193 529 [69.9%] non-Hispanic White, 13 611 [4.9%] non-Hispanic multiracial or other race) had nonmissing exposure data and were included in the analytic sample. After applying survey weights, this sample represents approximately 35 583 145 US households with children. Among this weighted sample, 580 000 (1.6%; 95% CI, 1.5%-1.8%) reported displacement in the past year. Displaced households were more likely to be Hispanic or Black, low income, and located in the South compared with nondisplaced households (Table 1). The most common identified disasters resulting in displacement were hurricanes (150 000 [25.9%]).

**Table 1.** Weighted Sample Characteristics of US Households With Children by Displacement Status^a^

Characteristic	Households, No. (%)^b^		*P* value
	Not displaced (n = 35 000 000)	Displaced (n = 580 000)	
Age, mean (SD), y	42.70 (0.05)	43.50 (0.46)	<.001
Sex			
Male	16 000 000 (44.4)	240 000 (41.0)	.04
Female	19 000 000 (55.6)	350 000 (59.0)	
Race and ethnicity^c^			
Hispanic	7 100 000 (20.2)	140 000 (24.7)	<.001
Non-Hispanic Asian	1 900 000 (5.5)	27 000 (4.7)	
Non-Hispanic Black	4 700 000 (13.5)	160 000 (27.4)	
Non-Hispanic White	20 000 000 (56.1)	210 000 (36.6)	
Non-Hispanic other or multiracial^d^	1 700 000 (4.7)	39 000 (6.6)	
Marital status			
Never married	6 700 000 (19.1)	160 000 (28.0)	<.001
Widowed, separated or divorced	5 900 000 (16.8)	140 000 (24.4)	
Married or domestic partnership	22 000 000 (63.8)	280 000 (47.3)	
Missing	100 000 (0.3)	2000 (0.3)	
Employment status			
Employed	25 000 000 (70.4)	350 000 (60.1)	<.001
Missing	190 000 (0.6)	9000 (1.5)	
Household job loss			
Recent household job loss	4 800 000 (13.7)	190 000 (32.0)	<.001
Missing	68 000 (0.19)	2000 (0.3)	
Disability status			
At least 1 disability	3 700 000 (10.7)	130 000 (22.8)	<.001
Missing	970 000 (2.8)	25 000 (4.3)	
Educational status			
Less than high school	2 900 000 (8.2)	99 000 (17.0)	<.001
High school or equivalent	9 800 000 (27.9)	160 000 (27.3)	
Some college or more	22 000 000 (63.9)	330 000 (55.7)	
Household income, $			
<35 000	7 000 000 (20.1)	200 000 (34.7)	<.001
35 000-74 999	8 600 000 (24.6)	130 000 (22.7)	
75 000 -149 999	9 200 000 (26.4)	94 000 (16.1)	
≥150 000	6 700 000 (19.2)	75 000 (12.8)	
Missing	3 400 000 (9.8)	80 000 (13.7)	
Ownership and payment status			
Renter, caught up	8 300 000 (23.8)	120 000 (19.8)	<.001
Renter, behind	1 900 000 (5.4)	78 000 (13.4)	
Owner, caught up	22 000 000 (61.5)	270 000 (45.8)	
Owner, behind	1 200 000 (3.6)	32 000 (5.5)	
Not applicable	530 000 (1.5)	35 000 (6.1)	
Missing	1 500 000 (4.3)	55 000 (9.4)	
No. of children in home			
1	16 000 000 (44.3)	220 000 (37.2)	<.001
2	12 000 000 (34.6)	180 000 (31.5)	
≥3	7 400 000 (21.1)	180 000 (31.3)	
Total household size			
2	2 900 000 (8.3)	62 000 (10.5)	<.001
3-4 people	21 000 000 (59.2)	300 000 (51.1)	
≥5	11 000 000 (32.5)	220 000 (38.4)	
Energy insecurity			
Energy insecurity	16 000 000 (46.8)	380 000 (65.3)	<.001
Missing	2 000 000 (5.7)	60 000 (10.3)	
Food insufficiency			
Food insufficiency	4 800 000 (13.7)	200 000 (33.4)	<.001
Missing	140 000 (0.4)	7000 (1.2)	
Insurance type			
Public	10 000 000 (29.8)	190 000 (37.5)	<.001
Private	18 000 000 (52.4)	220 000 (32.3)	
Other	3 800 000 (10.8)	110 000 (18.9)	
Missing	2 400 000 (7.0)	66 000 (11.4)	
Adult mental health			
Depression or anxiety	22 000 000 (61.5)	430 000 (72.9)	<.001
Missing	500 000 (1.4)	17 000 (3.0)	
Disaster type			
Hurricane	NA	150 000 (24.9)	NA
Flood	NA	130 000 (21.9)	
Fire	NA	62 000 (10.7)	
Tornado	NA	85 000 (14.6)	
Other	NA	140 000 (24.7)	
Unknown	NA	19 000 (3.2)	
US region			
Northeast	5 800 000 (16.6)	60 000 (10.3)	<.001
South	14 000 000 (39.6)	350 000 (59.8)	
Midwest	7 400 000 (21.1)	80 000 (13.7)	
West	8 000 000 (22.8)	94 000 (16.2)	
Children in need of mental health care			
None	28 000 000 (81.2)	430 000 (73.2)	<.001
Some or all	5 400 000 (15.4)	130 000 (21.7)	
Missing	35 000 000 (3.4)	30 000 (5.1)	

Abbreviation: NA, not applicable.

^a^ Weighted sample of 35 583 145 households.

^b^ Unless otherwise indicated.

^c^ Self-reported by participants.

^d^ Other includes American Indian or Alaska Native, Native Hawaiian, Chamorro, Samoan, and Other Pacific Islander.

Displaced households were more likely to report pediatric mental health needs compared with nondisplaced households (odds ratio [OR], 1.29; 95% CI, 1.12-1.48) (Table 2). The adjusted predicted probability of pediatric mental health need was higher among displaced households (26.8%) than for nondisplaced households (18.8%). Among households with reported pediatric mental health needs, displaced households were more likely to report inadequate or no treatment, even after controlling for socioeconomic hardships (OR, 0.55; 95% CI, 0.38-0.66; *P* < .001). Households with incomes more than $150 000 were more likely to report mental health needs compared with households with less than $35 000 (OR, 1.24; 95% CI, 1.15-1.34; *P* < .001). Compared with non-Hispanic White respondents, non-Hispanic Asian (OR, 0.51; 95% CI, 0.47-0.56; *P* < .001), Black (OR, 0.58; 95% CI, 0.55-0.62; *P* < .001), and Hispanic (OR, 0.83; 95% CI, 0.78-0.88; *P* < .001) households were significantly less likely to report child mental health needs. Households with an adult reporting symptoms of depression and/or anxiety were more likely to report pediatric mental health need than households in which the adult did not have depressive or anxious symptoms (OR, 1.86; 95% CI, 1.79-1.94, *P* < .001). Households with material hardship, such as food insufficiency (OR, 1.16; 95% CI, 1.09-1.23; *P* < .001) and energy insecurity (OR, 1.17; 95% CI, 1.12-1.21; *P* < .001), were also more likely to have mental health needs compared with those without.

**Table 2.** ORs for Child Mental Health Needs by Disaster Displacement Status^a^

Variable	OR (95% CI) [SE]	*P* value
Displacement status		
No, not displaced	1.0 [Reference]	NA
Yes, displaced	1.29 (1.12-1.48) [0.09]	.001
Age		
Age (linear)	1.06 (1.05-1.08) [0.01]	<.001
Age squared (quadratic)	1.00 (1.00-1.00) [0.00]	<.001
Sex		
Male	1.0 [Reference]	NA
Female	1.29 (1.24-1.33) [0.03]	<.001
Race and ethnicity		
Hispanic	0.83 (0.78-0.88) [0.24]	<.001
Non-Hispanic Asian	0.51 (0.47-0.56) [0.02]	<.001
Non-Hispanic Black	0.58 (0.55-0.62) [0.18]	<.001
Non-Hispanic White	1.0 [Reference]	NA
Non-Hispanic multiracial or other^b^	0.90 (0.84-0.97) [0.03]	.004
Marital status		
Married or domestic partnership	1.0 [Reference]	NA
Never married	1.36 (1.28-1.45) [0.04]	<.001
Widowed, separated, or divorced	1.33 (1.26-1.45) [0.03]	<.001
Employment status		
Unemployed	1.0 [Reference]	NA
Employed	1.09 (1.04-1.14) [0.03]	<.001
Recent household job loss		
No recent job loss	1.0 [Reference]	NA
Recent household job loss	1.12 (1.05-1.18) [0.03]	<.001
Disability status		
No disability	1.0 [Reference]	NA
≥1 Disabilities	1.56 (1.48-1.65) [0.04]	<.001
Educational status		
Less than high school	1.0 [Reference]	NA
High school or equivalent	0.95 (0.85-1.06) [0.05]	.35
Some college or higher	1.24 (1.10-1.34) [0.06]	<.001
Household income, $		
<35 000	1.0 [Reference]	NA
35 000 -74 999	0.98 (0.92-1.05) [0.03]	.59
75 000-149 999	1.04 (0.97-1.12) [0.04]	.27
≥150 000	1.24 (1.15-1.34) [0.05]	<.001
Ownership and payment status		
Owner caught up	1.0 [Reference]	NA
Owner behind	1.06 (0.97-1.16) [0.05]	.18
Renter caught up	0.98 (0.93-1.03) [0.03]	.37
Renter behind	1.07 (0.97-1.19) [0.06]	.19
NA or occupied without payment	1.09 (0.95-1.25) [0.08]	.23
Total household size		
2	1.0 [Reference]	NA
3-4	0.75 (0.70-0.80) [0.03]	<.001
≥5	0.81 (0.74-0.87) [0.03]	<.001
No. of children in household		
1	1.0 [Reference]	NA
2	1.39 (1.33-1.45) [0.03]	<.001
≥3	1.69 (1.59-1.80) [0.06]	<.001
Food insufficiency		
Food sufficient	1.0 [Reference]	NA
Food insufficiency	1.16 (1.09-1.23) [0.03]	<.001
Energy insecurity		
Energy secure	1.0 [Reference]	NA
Energy insecurity	1.17 (1.12-1.21) [0.02]	<.001
Anxiety or depression		
No symptoms	1.0 [Reference]	NA
Symptoms of anxiety or depression	1.87 (1.79-1.94) [0.04]	<.001
Insurance type		
Private	1.0 [Reference]	NA
Public	1.21 (1.16-1.27) [0.03]	<.001
Other	0.93 (0.86-1.00) [0.04]	.07

Abbreviations: NA, not applicable; OR, odds ratio.

^a^ Models included indicator categories for missing covariate values to retain observations with incomplete covariate data; ORs for missing categories are not shown.

^b^ Other includes American Indian or Alaska Native, Native Hawaiian, Chamorro, Samoan, and Other Pacific Islander.

### Receipt of Pediatric Mental Health Treatment

In the sample, 51 143 households reported pediatric mental health needs. For this subsequent model, Vermont cases (n = 612) were excluded due to missing mental health practitioner data, yielding an analytic sample of 50 548 households representing 5 485 193 survey-weighted US households (Table 3). A total of 811 households reported displacement, whereas 49 737 did not. Among households with children, those that experienced displacement due to natural disaster had a significantly higher chance of reporting that at least 1 child needed mental health counseling or medication (OR, 1.29; 95% CI, 1.12-1.48; *P* < .001). Among displaced households that needed mental health care, 70 000 (55.1%) reported receipt of some or none. Among households with children who needed mental health treatment, those that experienced disaster-related displacement were less likely to receive all mental health treatment needed compared with nondisplaced households (OR, 0.55; 95% CI, 0.38-0.66; *P* < .001) (Table 4). Children in displaced households who needed mental health care were significantly less likely to receive it, with an adjusted receipt probability of 51.0% compared with 75.6% among nondisplaced households. Black households were less likely to receive complete mental health treatment for children in need compared with White households (OR, 0.42; 95% CI, 0.37-0.48; *P* < .001). Households with annual incomes of more than $150 000 were more likely to receive care compared with those with low incomes (OR, 1.53; 95% CI, 1.29-1.81; *P* < .001). Households with material hardship, such as food insufficiency (OR, 0.73; 95% CI, 0.66-0.80; *P* < .001), energy insecurity (OR, 0.73; 95% CI, 0.66-0.80; *P* < .001), or being behind on mortgage or rent (OR, 0.78; 95% CI, 0.64-0.96; *P* = .02), also were less likely to receive mental health treatment when compared with those without hardship.

**Table 3.** Weighted Sample Characteristics of Households With Pediatric Mental Health Needs by Displacement Status^a^

Characteristic	Households, No. (%)^b^		*P* value
	Not displaced (n = 5 400 000)	Displaced (n = 130 000)	
Age, mean (SD), y	43.82 (0.11)	45.91 (0.91)	<.001
Sex			
Male	2 000 000 (36.5)	48 000 (37.7)	.68
Female	3 400 000 (63.5)	79 000 (62.3)	
Race and ethnicity^c^			
Hispanic	930 000 (17.3)	35 000 (27.5)	<.001
Non-Hispanic Asian	140 000 (2.7)	50 000 (39.1)	
Non-Hispanic Black	560 000 (10.5)	26 000 (20.8)	
Non-Hispanic White	3 400 000 (63.8)	3199 (2.5)	
Non-Hispanic multiracial or other^d^	300 000 (5.7)	13 000 (10.1)	
Marital status			
Never married	980 000 (18.2)	39 000 (30.1)	<.001
Widowed, separated, or divorced	1 200 000 (22.8)	41 000 (32.6)	
Married or domestic partnership	3 100 000 (58.7)	46 000 (36.2)	
Missing	17 000 (0.3)	436 (0.3)	
Employment status			
Employed	3 800 000 (71.0)	78 000 (61.9)	<.001
Missing	15 000 (0.3)	1600 (1.3)	
Household job loss			
Recent household job loss	860 000 (16.1)	54 000 (42.3)	<.001
Missing	11 000 (0.20)	142 (0.11)	
Disability status			
≥1 Disabilities	960 000 (17.9)	52 000 (40.9)	<.001
Missing	110 000 (2.0)	5000 (4.0)	
Educational status			
Less than high school	390 000 (7.3)	27 000 (21.4)	<.001
High school or equivalent	1 300 000 (23.6)	30 000 (23.8)	
Some college or more	3 700 000 (69.1)	70 000 (54.8)	
Household income, $			
<35 000	1 200 000 (22.3)	48 000 (37.7)	<.001
35 000 -74 999	1 300 000 (24.5)	27 000 (21.4)	
75 000-149 999	1 400 000 (25.9)	20 000 (16.0)	
≥150 000	1 100 000 (20.3)	20 000 (16.0)	
Missing	380 000 (7.1)	12 000 (9.4)	
Ownership and payment status			
Renter, caught up	1 200 000 (23.0)	28 000 (21.7)	<.001
Renter, behind	350 000 (6.5)	15 000 (12.0)	
Owner, caught up	3 300 000 (61.1)	51 000 (40.4)	
Owner, behind	240 000 (4.5)	8600 (6.8)	
Not applicable	90 000 (1.7)	16 000 (12.4)	
Missing	170 000 (3.2)	8500 (6.7)	
No. of children in home			
1	1 900 000 (35.7)	33 000 (26.0)	<.001
2	2 000 000 (37.0)	34 000 (26.9)	
≥3	1 500 000 (27.3)	60 000 (47.2)	
Total household size			
2	380 000 (7.2)	8600 (6.8)	.002
3-4	2 900 000 (53.8)	55 000 (43.4)	
≥5	2 100 000 (39.1)	63 000 (49.9)	
Energy insecurity			
Energy insecurity	2 900 000 (54.9)	99 000 (77.7)	<.001
Missing	210 000 (4.0)	8900 (7.0)	
Food insufficiency			
Food insufficiency	960 000 (17.9)	56 000 (43.6)	<.001
Missing	14 000 (0.3)	800 (0.6)	
Insurance type			
Public	1 900 000 (36.1)	39 000 (30.5)	<.001
Private	2 700 000 (50.1)	48 000 (37.8)	
Other	480 000 (9.0)	31 000 (24.2)	
Missing	260 000 (4.8)	9500 (7.5)	
Adult mental health			
Depression or anxiety	4 200 000 (78.0)	110 000 (89.2)	<.001
Missing	17 000 (0.3)	600 (0.5)	
Disaster type			
Hurricane	NA	21 000 (16.9)	NA
Flood	NA	29 000 (22.5)	
Fire	NA	18 000 (14.4)	
Tornado	NA	19 000 (14.7)	
Other	NA	39 000 (29.0)	
Unknown	NA	2100 (2.4)	
US region			
Northeast	920 000 (17.1)	15 000 (12.1)	.001
South	2 000 000 (37.3)	62 000 (49.1)	
Midwest	1 300 000 (23.4)	23 000 (17.8)	
West	1 200 000 (22.2)	27 000 (21.0)	
Mental health treatment			
Needed but received some or no care	1 400 000 (26.0)	70 000 (55.1)	<.001
Needed and received all care	3 900 000 (73.1)	56 000 (43.9)	
Missing	48 000 (0.9)	1300 (1.0)	
Mental health professional shortage area			
Lowest unmet need	1 100 000 (20.1)	18 000 (14.3)	<.001
Low-moderate unmet need	1 700 000 (31.5)	41 000 (32.7)	
Moderate-high unmet need	1 400 000 (25.8)	45 000 (35.7)	
Highest unmet need	1 200 000 (22.7)	22 000 (17.3)	

Abbreviation: NA, not applicable.

^a^ Weighted sample of 5 485 193 households.

^b^ Unless otherwise indicated.

^c^ Self-reported by participants.

^d^ Other includes American Indian or Alaska Native, Native Hawaiian, Chamorro, Samoan, and Other Pacific Islander.

**Table 4.** ORs of Receiving Mental Health Treatment Among Children With Needs by Displacement Status^a^

Variable	OR (95% CI) [SE]	*P* value
Displacement status		
No, not displaced	1.0 [Reference]	NA
Yes, displaced	0.55 (0.38-0.66) [0.07]	<.001
Age		
Age (linear)	1.00 (0.98-1.02) [0.01]	.97
Age squared (quadratic)	1.00 (1.00-1.00) [0.00]	.47
Sex		
Male	1.0 [Reference]	NA
Female	0.96 (0.88-1.05) [0.04]	.34
Race and ethnicity		
Hispanic	0.58 (0.51-0.65) [0.03]	<.001
Non-Hispanic Asian	0.39 (0.37-0.48) [0.04]	<.001
Non-Hispanic Black	0.42 (0.37-0.48) [0.03]	<.001
Non-Hispanic White	1.0 Reference	NA
Non-Hispanic multiracial or other^b^	0.61 (0.53-0.71) [0.04]	<.001
Marital status		
Married or domestic partnership	1.0 [Reference]	NA
Never married	0.88 (0.77-1.01) [0.06]	.08
Widowed, separated, or divorced	0.91 (0.82-1.01) [0.05]	.08
Employment status		
Unemployed	1.0 [Reference]	NA
Employed	0.95 (0.86-1.05) [0.05]	.34
Recent household job loss		
No recent job loss	1.0 [Reference]	NA
Recent household job loss	0.76 (0.68-0.85) [0.04]	<.001
Educational status		
Less than high school	1.0 [Reference]	NA
High school or equivalent	1.19 (0.96-1.48) [0.13]	.11
Some college or higher	1.25 (1.03-1.52) [0.12]	.02
Household income, $		
<35 000	1.0 [Reference]	NA
35 000 -74 999	1.07 (0.94-1.22) [0.07]	.32
75 000-149 999	1.09 (0.95-1.26) [0.08]	.23
≥150 000	1.53 (1.29-1.81) [0.13]	<.001
Ownership and payment status		
Owner caught up	1.0 [Reference]	NA
Owner behind	0.80 (0.67-0.96) [0.07]	.02
Renter caught up	0.87 (0.79-0.97) [0.05]	.01
Renter behind	0.78 (0.64-0.96) [0.08]	.02
NA or occupied without payment	0.58 (0.45-0.76) [0.08]	<.001
Total household size		
2	1.0 [Reference]	NA
3-4	0.83 (0.69-0.99) [0.08]	.04
≥5	0.63 (0.52-0.77) [0.06]	<.001
No. of children in household		
1	1.0 [Reference]	NA
2	0.77 (0.70-0.85) [0.04]	<.001
≥3	0.73 (0.64-0.83) [0.05]	<.001
Food insufficiency		
Food sufficient	1.0 [Reference]	NA
Food insufficiency	0.65 (0.58-0.72) [0.04]	<.001
Energy insecurity		
Energy secure	1.0 [Reference]	NA
Energy insecurity	0.73 (0.66-0.80) [0.04]	<.001
Disability status		
No disability	1.0 [Reference]	NA
≥1 Disabilities	0.82 (0.74-0.91) [0.04]	<.001
Anxiety or depression		
No symptoms	1.0 [Reference]	NA
Symptoms of anxiety or depression	0.63 (0.56-0.71) [0.04]	<.001
Insurance type		
Private	1.0 [Reference]	NA
Public	1.15 (1.04-1.27) [0.06]	.008
Other	0.73 (0.62-0.85) [0.06]	<.001
Mental health professional shortage scarcity		
Lowest unmet need	1.0 [Reference]	NA
Low-moderate unmet need	0.85 (0.58-1.26) [0.17]	.42
Moderate-high unmet need	1.03 (0.69-1.54) [0.21]	.88
Highest unmet need	1.37 (0.87-2.14) [0.31]	.17

Abbreviations: NA, not applicable; OR, odd ratio.

^a^ Models included indicator categories for missing covariate values to retain observations with incomplete covariate data; ORs for missing categories are not shown.

^b^ Other includes American Indian or Alaska Native, Native Hawaiian, Chamorro, Samoan, and Other Pacific Islander.

### Sensitivity Analyses

Our sensitivity analysis assessed a less strict receipt of treatment definition (any care received coded as yes). This sensitivity analysis did not show statistically significantly lower odds of care for displaced households compared with nondisplaced households (OR, 0.75; 95% CI, 0.56-1.00; *P* = .056).

### Supplemental Analyses

#### Comparison of Excluded and Included Data

We calculated descriptive statistics to compare households with children included in the analytic sample (n = 277 081) with those excluded due to missing exposure data (n = 47 385) during the study period to assess potential selection bias introduced by missingness (eTable 3 in Supplement 1). The excluded population was more likely to have missing socioeconomic data compared with the analytic sample. For example, weighted analyses show that rates of missing household income data were significantly higher among excluded respondents (8 100 000 [98.9%]) than included respondents (36 000 000 [9.8%]) as seen in eTable 3 in Supplement 1.

#### Effect Modification Analysis

The interaction terms between displacement and income were not statistically significant (eTable 4 in Supplement 1), suggesting that the association between displacement and receipt of mental health treatment did not meaningfully differ by income group. The interaction terms with mental health practitioner scarcity and displacement status were also not statistically significant (eTable 5 in Supplement 1).

## Discussion

This investigation is one of the first explorations of mental health needs of children and receipt of care among disaster-displaced households. Using survey weights, we estimate that more than 500 000 households with children were displaced because of natural disasters since 2023. Our findings suggest that displaced families report higher need for pediatric mental health treatment, yet these needs are less likely to be met compared with children in nondisplaced households.

### Mental Health Needs Among Displaced Children

A recent review^17^ on climate change and children’s well-being found that most research has examined posttraumatic stress disorders after climate disasters, whereas depression and anxiety remain understudied. A scoping review^18^ highlighted an urgent need to better understand the impacts of climate-related disasters on mental disorders among at-risk children. A few smaller studies^19,20,21^ of displaced families impacted by Hurricane Katrina found elevated mental health concerns and limited access to services. Our findings add to this increasing body of literature suggesting that these broader mental health aftershocks, especially among the increasing population of displaced children in the US, warrant deeper investigation.

### Barriers to Mental Health Care Access

Our results call attention to potential barriers to mental health care access in households of color and low-income households, possibly due to historical, cultural, and structural factors. This aligns with prior studies^22,23,24^ that found that families of color tend to underrecognize pediatric mental health needs, whereas higher-income households are more likely to report them. Even when low-income households of color share concern for mental health needs, these children are less likely to receive all needed care. This finding underscores preexisting racial and ethnic disparities documented in prior literature.^15,16^ Elliot et al^15^ and Annis et al^16^ have documented profound disparities in mental health needs and access to care among children of different races and ethnicities, exposing underlying vulnerable fault lines regarding pediatric mental health.

These racial and ethnic disparities in treatment access experienced by displaced individuals are likely driven by interconnected structural and socioeconomic barriers, including housing instability, transportation limitations, financial insecurity, fractured health care systems, and other competing priorities. Although accounting for socioeconomic factors in our analyses, displacement remains a driving force behind unmet pediatric mental health care needs demonstrating that displacement likely introduces distinct disruptions beyond socioeconomic pressures. Additionally, our supplemental analysis comparing the excluded cases to the analytic sample suggests that complete case analysis may underestimate unmet mental health care needs. Excluded households had higher rates of missing socioeconomic data, which may correlate with unmeasured socioeconomic disadvantage.^25^

### Health System and Policy Considerations

Health care professionals and policymakers should ensure that disaster preparedness and response teams prioritize mental well-being and access to mental health treatment for households with children. This is certainly a challenge in a health care system, which already has long waitlists and limited pediatric mental health practitioners.^26^ With this context, our results show persistent lack of treatment regardless of mental health practitioner quartile. Mental health support for children after disaster can be especially difficult, but studies^27,28,29^ have acknowledged schools as a promising site for recovery and intervention efforts. As Fortuna and colleagues^30^ have recommended, strategies for mitigating the mental health effects of climate-related displacement include cultivating supportive and communal responses. School-based mental health initiatives, trauma-informed parenting support, or accessible child psychology services should be prioritized in disaster-affected areas. Natural disasters have longstanding consequences and policies need to consider long-term recovery and a wide spectrum of support, not just immediate relief or narrow focus on diagnoses such as posttraumatic stress disorder. This entails sustained funding and access to mental health treatment for months or even years after an event to mitigate health care disruption and promote resilience in youth affected by natural disasters. The Federal Emergency Management Agency should also consider embedding pediatric behavioral health in its recovery frameworks. More broadly, the substantial burden of mental health concerns and limited receipt of care among both displaced and nondisplaced households reinforces the need to invest in and strengthen our mental health infrastructure to ensure access to screening and treatment for all children.

### Limitations

Limitations of this study include its cross-sectional design and reliance on self-reported data, limiting causality and temporality inferences. There may also be a self-selection bias in responding. Due to measuring displacement in the past year and mental health needs in the past 4 weeks, temporal overlap cannot be established because some reported needs may predate the disaster. Additionally, the database lacks potential factors that may affect access to mental health, such as preferred language, health care accessibility, granular child-specific sociodemographic data, and survey respondent relationship to household children. The need for mental health treatment may possibly reflect caregiver opinion without health care professional assessment and input. Furthermore, displacement may capture cumulative vulnerability, and its duration is not assessed. Additionally, the proxy for mental health care access did not account for psychologists, therapists, social workers, or nurses. Although complete-case analysis may introduce bias, inverse probability weighting was not feasible due to pervasive and likely nonrandom missingness in key covariates. Despite limitations, these results should inform development of data collection tools that distinguish disaster exposure from displacement and obtain more objective and granular data. Although the magnitude and significance of the association between displacement and receipt of needed mental health care were attenuated in the sensitivity analysis, the direction of the result remained consistent, which warrants further study.

## Conclusions

This cross-sectional study found that among households with children, those that were displaced due to natural disaster had higher odds of reporting a need for pediatric mental health care compared with nondisplaced households. These findings suggest a gap in pediatric mental health care among disaster-displaced households, underscoring the need for equity-driven, child-centered responses in climate and disaster preparedness.

## Notes

Supplement 1. **eFigure.** Derivation of the Analytic Sample **eTable 1.** Household Pulse Survey Variable Categorization **eTable 2.** Disaster-Related Displacement Exposure and Outcome Definitions Across Analytic Models **eTable 3.** Comparison of Excluded vs Included Households With Children **eTable 4.** Association Between Disaster Displacement and Receipt of Mental Health Care, by Income (N = 50548) **eTable 5.** Association Between Disaster Displacement and Receipt of Mental Health Care, by Unmet Need Quartile (N = 50548)

Supplement 2.
Data Sharing Statement
