# Supplementary material for: Pediatric Mental Health Needs, Unmet Care, and Disaster-Related Displacement
Source: JAMA Netw Open. 2026 Apr 14;9(4):e264922. doi: 10.1001/jamanetworkopen.2026.4922 (PMC13080542; doi:10.1001/jamanetworkopen.2026.4922)
Supplement: Supplement 1. — eFigure. Derivation of the Analytic Sample eTable 1. Household Pulse Survey Variable Categorization eTable 2. Disaster-Related Displacement Exposure and Outcome Definitions Across Analytic Models eTable 3. Comparison of Excluded vs Included Households With Children eTable 4. Association Between Disaster Displacement and Receipt of Mental Health Care, by Income (N = 50548) eTable 5. Association Between Disaster Displacement and Receipt of Mental Health Care, by Unmet Need Quartile (N = 50548) [file jamanetwopen-e264922-s001.pdf]

Supplemental Online Content

Ceasar JN, Cabrera KI, Mandell D. Pediatric mental health needs, unmet care, and disaster-related displacement. *JAMA Netw Open*. 2026;9(4):e264922.
doi:10.1001/jamanetworkopen.2026.4922

eFigure. Derivation of the Analytic Sample

eTable 1. Household Pulse Survey Variable Categorization

eTable 2. Disaster-Related Displacement Exposure and Outcome Definitions Across Analytic Models

eTable 3. Comparison of Excluded vs Included Households With Children[ab]

eTable 4. Association Between Disaster Displacement and Receipt of Mental Health Care, by Income (N = 50548)

eTable 5. Association Between Disaster Displacement and Receipt of Mental Health Care, by Unmet Need Quartile (N = 50548)

This supplemental material has been provided by the authors to give readers additional information about their work.

© 2026 Ceasar JN et al. *JAMA Network Open*.

eFigure. Derivation of the Analytic Sample

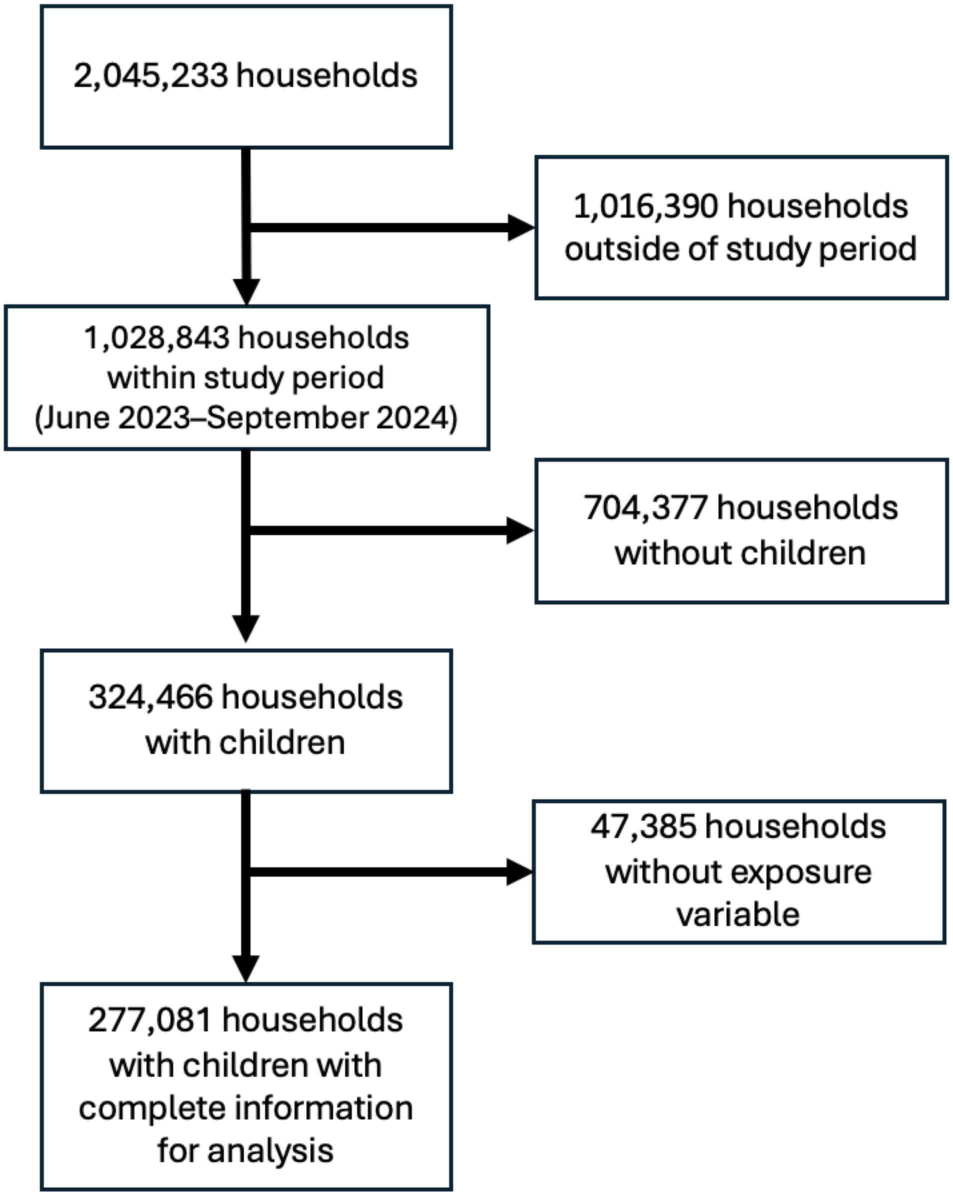

Flow diagram showing derivation of the analytic sample from the U.S. Census Bureau Household Pulse Survey. Households were restricted to the study period (week 58–cycle 9), then to those with children, and further to those with complete data on the exposure of displacement, yielding a final analytic sample of 277,081 households.

© 2026 Ceasar JN et al. *JAMA Network Open*.

eTable 1. Household Pulse Survey Variable Categorization

Variable type	Variable name	Survey item	Survey answer choices	New variable categories
**Exposure**	Displacement	Have you or has anyone in your household had to move from your home in the last 12 months because of a natural disaster?	1) Yes<br>2) No	Yes or No
**Outcomes**	Pediatric mental health needs	During the last 4 weeks, did any children in your household need mental health treatment? Mental health treatment includes health services like counseling or medication.	1) Yes, all children needed mental health treatment<br>2) Yes, some but not all children needed mental health treatment<br>3) No, none of the children needed mental health treatment	Yes if answered ‘yes, all children needed mental health treatment’ or ‘yes, some children needed mental health treatment’<br><br>No if answered ‘no, none of the children needed mental health treatment’.
	Pediatric mental health treatment receipt (main model)	Did the children who needed mental health treatment receive it?	1) Yes, all children who needed treatment received it<br>2) Yes, but only some children who needed treatment received it<br>3) No, none of the children who needed treatment received it	Yes if answered ‘all children received treatment’<br><br>No if answered ‘no, none’ or ‘only some children received treatment’

© 2026 Ceasar JN et al. *JAMA Network Open*.

	Pediatric mental health treatment receipt (sensitivity analysis)	Did the children who needed mental health treatment receive it?	1) Yes, all children who needed treatment received it<br>2) Yes, but only some children who needed treatment received it<br>3) No, none of the children who needed treatment received it	Yes if answered 'all or some children received treatment'<br><br>No if answered 'no, none'
**Socio-demographic covariates**	Age	What year were you born? Please enter a number.	Numerical answer	Age of head of household in years (continuous)
	Female	What sex were you assigned at birth, on your original birth certificate?	1) Male<br>2) Female	Yes if answered 'female'<br><br>No if answered 'male'
	Race/ethnicity	Are you of Hispanic, Latino, or Spanish origin?	1) No, not of Hispanic, Latino, or Spanish origin<br>2) Yes, of Hispanic, Latino, or Spanish origin	Hispanic<br><br>Non-Hispanic<br><br>Non-Hispanic Asian<br><br>Non-Hispanic Black<br><br>Non-Hispanic White<br><br>Other/Mixed
		What is your race? Please select all that apply	1) White, Alone<br>2) Black, Alone<br>3) Asian, Alone<br>4) Any other race alone, or race in combination[a]	

© 2026 Ceasar JN et al. *JAMA Network Open*.

	Educational attainment	What is the highest degree or level of school you have completed? Select only one answer.	1) Less than high school<br>2) Some high school<br>3) High school graduate or equivalent (for example GED)<br>4) Some college, but degree not received or is in progress<br>5) Associate’s degree (for example AA, AS)<br>6) Bachelor's degree (for example BA, BS, AB)<br>7) Graduate degree (for example master's, professional, doctorate)	Less than high school<br><br>High school or equivalent<br><br>Some college or higher
	Employment status	Now we are going to ask about your employment. In the last 7 days, did you do ANY work for either pay or profit? Select only one answer.	1) Yes<br>2) No	Yes<br><br>No<br><br>Missing
	Household job loss	Have you, or has anyone in your household experienced a loss of employment	1) Yes<br>2) No	Yes<br><br>No<br><br>Missing

© 2026 Ceasar JN et al. *JAMA Network Open*.

		income in the last 4 weeks? Select only one answer.		
	Marital status	What is your marital status? Select only one answer.	1) Now married<br>2) Widowed<br>3) Divorced<br>4) Separated<br>5) Never married	Now married<br><br>Never married<br><br>Widowed, divorced or separated<br><br>Missing
**Household characteristics**	Household income	In 2021 what was your total household income before taxes? Select only one answer.	1) Less than $25,000<br>2) $25,000 - $34,999<br>3) $35,000 - $49,999<br>4) $50,000 - $74,999<br>5) $75,000 - $99,999<br>6) $100,000 - $149,999<br>7) $150,000 - $199,999<br>8) $200,000 and above<br>-99) Question seen but category not selected<br>-88) Missing / Did not report	<$35,000<br><br>$35,000–$74,999<br><br>$75,000–$149,999<br><br>≥$150,000<br><br>Missing
	Total household size	How many total people – adults and children – currently live in your household, including yourself? Please enter a number.	Numerical answer	1<br>2<br>3–4<br>5 or more

© 2026 Ceasar JN et al. *JAMA Network Open*.

	Number of children	How many people under 18 years-old currently live in your household? Please enter a number.	Numerical answer	0<br>1<br>2<br>3 or more
	Housing security	Is your house or apartment…? Select only one answer.	1) Owned by you or someone in this household free and clear?<br>2) Owned by your or someone in this household with a mortgage or loan (including home equity loans)?<br>3) Rented?<br>4) Occupied without payment of rent?	Owner caught up on mortgage<br><br>Owner behind<br><br>Renter caught up on lease<br><br>Renter behind<br><br>Not applicable<br><br>Missing
		Is this household currently caught up on rent payments? Select only one answer. OR Is this household currently caught up on mortgage payments? Select only one answer.	1) Yes<br>2) No	

© 2026 Ceasar JN et al. *JAMA Network Open*.

	Food Insufficiency	Getting enough food can also be a problem for some people. In the last 7 days, which of these statements best describes the food eaten in your household?	1) Enough of the kinds of food (I/we) wanted to eat<br>2) Enough, but not always the kinds of food (I/we) wanted to eat<br>3) Sometimes not enough to eat<br>4) Often not enough to eat	Yes if answered 'sometimes not enough to eat' or 'often not enough to eat' in past 7 days<br><br>No if answered 'enough of the kinds of food (I/we) wanted to eat' or 'enough, but not always the kinds of food (I/we) wanted to eat'<br><br>Missing
	Energy Insecurity	In the last 12 months, how many months did your household reduce or forego expenses for basic household necessities, such as medicine or food, in order to pay an energy bill?	1) Almost every month<br>2) Some months<br>3) 1 or 2 months<br>4) Never	Yes if answered 'almost every month', 'some months', or '1 or 2 months' to at least one of the questions.<br><br>No if all three indicators were answered 'never'.<br><br>Missing
		In the last 12 months, how many months did your household keep your home at a temperature that you felt was unsafe or unhealthy?		
		In the last 12 months, how many times was your household unable to pay		

© 2026 Ceasar JN et al. *JAMA Network Open*.

		an energy bill or unable to pay the full bill amount?		
**Head of Household Health Information**	Caregiver disability status	Do you have difficulty seeing, even when wearing glasses? Select one.	1) No - no difficulty<br>2) Yes - some difficulty<br>3) Yes - a lot of difficulty<br>4) Cannot do at all	Yes if answered 'yes' or 'cannot do at all' to any of the questions<br><br>No if answered 'no – no difficulty'<br><br>Missing
		Do you have difficulty hearing, even when using a hearing aid? Select one.		
		Do you have difficulty walking or climbing stairs? Select one.		
		Do you have difficulty remembering or concentrating? Select one.		
	Adult depression/anxiety	Over the last 2 weeks, how often have you been bothered by feeling nervous, anxious, or on edge? Select only one answer.	1) Not at all<br>2) Several days<br>3) More than half the days<br>4) Nearly every day	Yes if answered with 'several days', 'more than half the days' or 'nearly every day' to any of the questions<br><br>No if 'not at all'<br><br>Missing
		Over the last 2 weeks, how often have you been bothered by the not being able to stop or control worrying?		

© 2026 Ceasar JN et al. *JAMA Network Open*.

		Select only one answer.		
		Over the last 2 weeks, how often have you been bothered by having little interest or pleasure in doing things? Select only one answer.		
		Over the last 2 weeks, how often have you been bothered by feeling down, depressed, or hopeless? Select only one answer.		
	Health insurance type	Are you currently covered by any of the following types of health insurance or health coverage plans? Mark Yes or No for each.	* Insurance through a current or former employer or union (through yourself or another family member)<br>* Insurance purchased directly from an insurance company, including marketplace coverage (through yourself or another family member)<br>* Medicare,	Private (employer or direct purchase)<br><br>Public/government (Medicare, Medicaid, Tricare, VA, Indian Health Service)<br><br>Other<br><br>Missing

© 2026 Ceasar JN et al. *JAMA Network Open*.

			for people 65 and older, or people with certain disabilities<br>* Medicaid, Medical Assistance, or any kind of government-assistance plan for those with low incomes or a disability<br>* TRICARE or other military health care<br>* VA (including those who have ever used or enrolled for VA health care)<br>* Indian Health Service<br>* Other	

a. The Household Pulse Survey does not provide more granular data regarding race or ethnicity within the 'Other' category.

© 2026 Ceasar JN et al. *JAMA Network Open*.

eTable 2. Disaster-Related Displacement Exposure and Outcome Definitions Across Analytic Models

Model	Exposure	Sample	Outcome	Covariates
**Model 1: Mental Health Needs**	Displacement (household report of disaster-related displacement; reference = non-displaced)	277,081 households with children	Pediatric Mental Health Needs	Age, race, ethnicity, marital status, employment status, recent household job loss, disability status, educational attainment, household income, ownership and mortgage/rent payment status, household size, number of children in household, food insufficiency, energy insecurity, adult anxiety and/or depression, and insurance type
**Model 2: Receipt of Care**		50,548 households who reported mental health needs	Receipt of Mental Health Treatment (Strict Definition): all children in household with identified need received treatment	Model 1 + mental health provider scarcity
**Sensitivity Analysis**			Receipt of Mental Health Treatment (Less Restrictive Definition): some or all children in household received treatment	

All analyses were survey-weighted logistic regressions which included state fixed effects.

© 2026 Ceasar JN et al. *JAMA Network Open*.

eTable 3. Comparison of Excluded vs Included Households With Children[ab]

Variable		Included (No.) %	Excluded (No.) %	*p* value	Unweighted Sample Count
Age, [Mean (SD)], y		42.7 (0.05)	41.3 (0.12)	<0.001	324 466
Sex	Male	15 790 000 (44.4)	3 784 000 (46.8)	0.0001	324 466
	Female	19 793 000 (55.6)	4 297 000 (53.2)		
Race and Ethnicity[c]	Hispanic	7 200 000 (20.3)	2 100 000 (25.5)	<0.001	324 466
	Non-Hispanic Asian	1 900 000 (5.5)	430 000 (5.4)		
	Non-Hispanic Black	4 900 000 (13.7)	1 500 000 (19.1)		
	Non-Hispanic White	20 000 000 (55.8)	3 700 000 (45.2)		
	Non-Hispanic multiracial or other[d]	1 700 000 (4.8)	380 000 (4.8)		
Marital Status	Never married	6 900 000 (19.3)	2 200 000 (26.9)	<0.001	324 466
	Widowed, separated or divorced	6 000 000 (16.9)	1 400 000 (17.3)		
	Married or domestic partnership	23 000 000 (63.6)	4 400 000 (53.9)		
	Missing	110 000 (0.3)	160 000 (1.9)		
Employment Status	Employed	25 000 000 (70.2)	3 700 000 (45.75)	<0.001	324 466
	Missing	200 000 (0.6)	1 800 000 (22.3)		
Household Job Loss	Recent household job loss	5 000 000 (14.0)	1 100 000 (13.2)	<0.001	324 466
	Missing	69 000 (0.2)	8 100 000 (21.5)		
Disability	≥1 Disabilities	3 900 000 (10.9)	380 000 (4.8)	<0.001	324 466
	Missing	990 000 (2.8)	5 000 000 (61.5)		
Education	Less than high school	3 000 000 (8.3)	1 200 000 (14.6)	<0.001	324 466
	High school or equivalent	9900000 (27.9)	2 800 000 (35.2)		

© 2026 Ceasar JN et al. *JAMA Network Open*.

	Some college or higher	23 000 000 (63.8)	4 100 000 (50.2)		
Household Income	<$35,000	7 200 000 (20.3)	35 000 (0.4)	<0.001	324 466
	$35,000 - 74,999	8 700 000 (24.6)	34 000 (0.3)		
	$75,000 - 149,999	9 300 000 (26.2)	17 000 (0.2)		
	≥$150,000	6 800 000 (19.1)	13 000 (0.2)		
	Missing	36 000 000 (9.8)	8 100 000 (98.9)		
Home and payment status	Renter, caught up	8 400 000 (23.7)	24 000 (0.3)	<0.001	324 466
	Renter, behind	2 000 000 (5.5)	7892 (0.1)		
	Owner, caught up	22 000 000 (61.2)	48 000 (0.6)		
	Owner, behind	1 300 000 (3.6)	5056 (0.1)		
	NA or occupied without payment	5 600 000 (1.6)	4251 (0.1)		
	Missing	1 600 000 (4.4)	8 000 000 (98.9)		
Number of children in household	1	16 000 000 (44.2)	3 500 000 (43.2)	<0.001	324 466
	2	12 000 000 (34.6)	2 600 000 (32.8)		
	≥3	7 600 000 (21.3)	1 900 000 (24.0)		
Total household size	2	3 000 000 (8.4)	760 000 (9.4)	<0.001	324 466
	3 – 4	21 000 000 (59.0)	4 400 000 (54.0)		
	≥5	12 000 000 (32.6)	3 000 000 (36.7)		
Energy Insecurity	Energy Insecurity	17 000 000 (47.1)	56 000 (0.7)	<0.001	324 466
	Missing	2 100 000 (5.8)	8 000 000 (98.9)		
Food Insufficiency	Food Insufficiency	5 000 000 (14.0)	320 000 (3.9)	<0.001	324 466
	Missing	140 000 (0.4)	6 500 000 (81.0)		
Insurance Type	Public	11 000 000 (29.8)	120 000 (1.8)	<0.001	324 466
	Private	19 000 000 (52.1)	140 000 (1.5)		

© 2026 Ceasar JN et al. *JAMA Network Open*.

	Other	3 900 000 (11.0)	68 000 (0.8)		
	Missing	2 500 000 (7.1)	7 700 000 (95.8)		
Adult Mental Health	Symptoms of Depression or Anxiety	22 000 000 (61.7)	1 400 000 (17.5)	<0.001	324 466
	Missing	510 000 (1.4)	5 500 000 (67.7)		
Disaster Type	Hurricane	150 000 (24.9)	N/A	N/A	3 352
	Flood	130 000 (21.9)			
	Fire	62 000 (10.7)			
	Tornado	85 000 (14.6)			
	Other	140 000 (24.8)			
	Unknown	19 000 (3.2)			
US Region	Northeast	5 900 000 (16.5)	1 400 000 (16.9)	<0.001	324 466
	South	14 000 000 (39.9)	3 400 000 (41.7)		
	Midwest	7 500 000 (21.0)	1 600 000 (19.4)		
	West	8 100 000 (22.7)	1 800 000 (22.0)		
Mental Health Needs	Child with Mental Health Needs	5 500 000 (15.5)	34 000 (4.2)	<0.001	324 466
	Missing	1 200 000 (3.4)	5 600 000 (69.9)		
Mental Health Treatment	Needed but received incomplete or none	1 500 000 (26.7)	100 000 (30.5)	<0.001	53 355
	Needed and received all care	4 000 000 (72.4)	180 000 (53.0)		
	Missing	4 900 (0.9)	5 600 (16.4)		

Abbreviations: NA, not applicable

a. Unweighted sample size of 324 466 features 277 081 households included in analytic sample and 47 385 excluded households due to missing data

© 2026 Ceasar JN et al. *JAMA Network Open*.

b. Sample size of 324 466 represents 43 664 948 US households after applying survey weights (35 583 145 in analytic sample and 8 081 803 excluded from analyses)
c. Self-reported by survey participants
d. Other includes American Indian or Alaska Native, Native Hawaiian, Chamorro, Samoan, and Other Pacific Islander

© 2026 Ceasar JN et al. *JAMA Network Open*.

eTable 4. Association Between Disaster Displacement and Receipt of Mental Health Care, by Unmet Need Quartile (N = 50548)

**Variable**	**Odds Ratio (95% CI)**	**Standard Error**	***p* value**
**Displaced Status**	0.44 (0.26 – 0.74)	0.12	0.002
**Quartile 2: Low-moderate Unmet Need**	0.85 (0.57 – 1.25)	0.17	0.40
**Quartile 3: Moderate-high Unmet Need**	1.03 (0.69 – 1.54)	0.21	0.87
**Quartile 4: Highest Unmet Need**	1.36 (0.87 – 2.13)	0.31	0.18
**Displaced x Quartile 2**	1.44 (0.76 – 2.71)	1.13	0.26
**Displaced x Quartile 3**	1.07 (0.52 – 2.20)	0.19	0.85
**Displaced x Quartile 4**	1.60 (0.75 – 3.30)	1.21	0.23

Model covariates include age, race, ethnicity, marital status, employment status, recent household job loss, disability status, educational attainment, household income, ownership and mortgage/rent payment status, household size, number of children in household, food insufficiency, energy insecurity, adult anxiety and/or depression, insurance type and mental health provider scarcity. Model also includes state fixed effects.

© 2026 Ceasar JN et al. *JAMA Network Open*.
