# Supplementary material for: Pediatric Mental Health Needs, Unmet Care, and Disaster-Related Displacement
Source: JAMA Netw Open. 2026 Apr 14;9(4):e264922. doi: 10.1001/jamanetworkopen.2026.4922 (PMC13080542; doi:10.1001/jamanetworkopen.2026.4922)
Supplement: Supplement 2. — Data Sharing Statement [file jamanetwopen-e264922-s002.pdf]

Data Sharing Statement

Ceasar. Pediatric Mental Health Needs, Unmet Care, and Disaster-Related Displacement. *JAMA Netw Open*. Published April 14, 2026. doi:10.1001/jamanetworkopen.2026.4922

Data
Data available: Yes
Data types: Data (not involving human participants), Data dictionary
How to access data: https://www.census.gov/programs-surveys/household-pulse-survey/data.html
When available: With publication

Supporting Documents
Document types: None

Additional Information
Who can access the data: https://www.census.gov/programs-surveys/household-pulse-survey/data.html
Types of analyses: https://www.census.gov/programs-surveys/household-pulse-survey/data.html
Mechanisms of data availability: https://www.census.gov/programs-surveys/household-pulse-survey/data.html
